# Virtual reality–guided mindfulness for chronic pain in cancer survivors: protocol for the virtual mind study—a single-group feasibility trial

**DOI:** 10.3389/fpain.2024.1291374

**Published:** 2024-04-04

**Authors:** Mohamad Baydoun, Zen Gajtani, Michaela Patton, Andrew McLennan, Stephen Cartwright, Linda E. Carlson

**Affiliations:** ^1^ Faculty of Nursing, University of Regina, Regina, SK, Canada; ^2^ Department of Oncology, Cumming School of Medicine, University of Calgary, Calgary, AB, Canada; ^3^ Department of Psychology, University of Calgary, Calgary, AB, Canada; ^4^ Department of Psychology, University of Regina, Regina, SK, Canada; ^5^ Centre for Simulation and Visualization, University of Calgary, Calgary, AB, Canada

**Keywords:** cancer pain, chronic pain, integrative oncology, mind-body intervention, mindfulness meditation, virtual reality

## Abstract

**Background:**

Chronic cancer-related pain (CRP) can have a significant negative impact on quality of life. Mindfulness is hypothesized to mitigate chronic CRP by regulating both physical and emotional resistance to pain. In recent years, there has been interest in the use of virtual reality (VR) to deliver mindfulness meditation. VR provides an immersive and engaging environment, which may enhance one's focused attention to present-moment experiences, potentially making mindfulness less effortful and more efficacious for individuals with chronic pain. There has been little research in this area for people with a history of cancer.

**Objective:**

The aim of this mixed methods study is to evaluate the feasibility of a VR-guided mindfulness (VRGM) intervention offered to adult cancer survivors with chronic CRP.

**Methods:**

This mixed methods feasibility study will employ a single-arm, pretest-posttest design with semistructured interviews. In total, 15 cancer survivors will be enrolled in a 6-week home-based intervention that consists of 10–15 min of daily VRGM practice. The primary outcome is feasibility as assessed by accrual rates, retention in the study, intervention adherence, questionnaire completion, and side effect rates. Participants will be assessed on psychosocial outcome measures (i.e., pain, sleep, depressive and anxiety symptoms, fatigue, quality of life, and mindfulness) before and after the intervention, and 6 weeks post intervention (follow-up). Changes in pain will be described in relation to levels of immersion and presence in the virtual environment, trait mindfulness, and amount of VRGM practice. Qualitative information will provide subjective detail on participants’ experience with VRGM to complement quantitative data. This study has been approved by the Health Research Ethics Board of Alberta Cancer Committee (HREBA.CC-20-0411).

**Conclusions:**

This novel intervention provides a potential alternative treatment to pharmacological pain management. Results from this study may inform future larger VGRM trials for chronic CRP to help reduce suffering in people with cancer. Study findings will be disseminated through open access publications, traditional conference presentations, professional cancer organizations, and social media platforms.

## 1 Introduction

### 1.1 Background

Improved survival owing to advances in cancer treatment has resulted in increased numbers of patients experiencing chronic pain (1), defined by the International Association for the Study of Pain as “persistent or recurrent pain lasting longer than 3 months” (2). The prevalence of chronic cancer-related pain (CRP) has been estimated at 30%–50% in patients undergoing cancer treatment and more than 70% in patients with advanced, metastatic, or terminal disease (3). CRP is often felt as moderate or severe. A 2016 systematic review on the prevalence of CRP (3) found that 38% of 32,261 patients across 52 studies reported at least moderate CRP, while a 2009 European survey study (4) indicated that of 5,084 adults with cancer, 56% reported moderate to severe CRP.

CRP can be due to the cancer itself or its treatment. CRP directly related to cancer can arise from tumor invasion that can affect the viscera, nerves, and bones (5). CRP related to treatment is generally due to peripheral neuropathy from chemotherapeutic agents (e.g., vincristine and taxane), radiotherapy-induced neural damage, and chronic postsurgical pain from mastectomy, amputation, or thoracotomy (5). Certain treatments for cancer, such as head and neck radiation or conditioning regimens administered prior to hematopoietic cell transplant, can also cause painful gastrointestinal mucositis (6).

CRP is associated with psychological distress and functional impairment. One study of 667 individuals with various cancer types found that pain, when present, interfered with participants’ daily activity and enjoyment of life to a moderate to severe extent (7). To this end, chronic pain research often emphasizes the importance of managing pain interference in addition to pain intensity in order to improve quality of life (8). Descriptive studies of women with breast cancer also link pain to poor emotional, physical, and social functioning, in addition to increased fatigue, anxiety, depression, and insomnia (9, 10). Studies also support the link between poor sleep and increased risk for chronic diseases and mortality (11, 12). Therefore, effective treatment of CRP can have broad positive effects on the health and well-being of patients with and survivors of cancer by improving other symptoms related to quality of life and morbidity.

Opioid analgesics are the standard of care for people with chronic CRP (13), but they are limited by negative side effects and neuroadaptations associated with long-term use, which reduces analgesic efficacy (14). Some psychological and physical therapies (e.g., cognitive behavioral therapy, hypnosis, and acupuncture) have shown the potential to reduce levels of CRP as a stand-alone treatment or an adjunct to pharmacological interventions (15, 16). In light of the high prevalence of CRP among patients with and survivors of cancer and the limitations of currently existing treatments, the establishment of safe and effective nonpharmacological interventions for chronic CRP management should be a research priority.

One style of training in mindfulness meditation is a psychological approach that involves directing focused attention to the internal and external experiences occurring in the present moment with an accepting attitude (17, 18). By bringing attention to the present moment, one can cultivate a nonjudgmental awareness to everyday situations that may be perceived as stressful (17). This is particularly important for people with chronic CRP, as pain persistence itself may cause stress (19). It has been hypothesized that mindfulness practice over time would enhance trait mindfulness (i.e., one’s predisposition to be mindful in daily life) and consequently potentially improve pain outcomes by regulating emotional reactions to pain and enhancing acceptance-related coping approaches (20, 21). The biological mechanism explaining this effect with the most supportive evidence is that endogenous opioid pathways mediate the analgesic effects of mindfulness on pain through orbitofrontal cortex activation and thalamic deactivation (22).

A pioneering 1985 single-group study of mindfulness-based stress reduction (MBSR) by Kabat-Zinn (23) showed a significant reduction in chronic pain among 90 patients with a variety of conditions, even up to 4 years post intervention (24). More recent randomized controlled trials (RCTs) have also supported the positive effects of mindfulness on chronic pain (25). For example, in a 2011 RCT of people with irritable bowel syndrome (IBS) (26), participants reported significant pain improvement following a mindfulness-based intervention, and benefits were maintained 3 months post intervention. Multivariate path analyses suggested that mindfulness practice mitigated pain through improving anxiety and emotional responses to IBS symptoms (26, 27). While very few mindfulness studies targeting pain have been conducted among cancer survivors, the evidence to date is promising with studies showing small to moderate effects (28). For example, in a 2016 RCT (29) comparing MBSR to an active control condition (psychoeducational support) for breast and colorectal cancer survivors, MBSR participants reported significant reduction in pain compared with control participants (Cohen *d *= 0.53).

In the past few years, there has been a growing interest in using virtual reality (VR), a computer-generated 360° representation of a virtual environment displayed through a headset as a medium to support mindfulness practice (30). The multisensory information and stereo-visual image that VR delivers are meant to create a sense of space and depth, which can help users become fully present and immersed in the virtual atmosphere (31). Immersion is the degree to which the technological features of VR systems deliver an extensive, inclusive, surrounding, and vivid illusion of reality to the senses of a human participant, whereas presence is the perception of being present in a VR environment (i.e., a feeling of being there) (32). Although other platforms that provide guidance on mindfulness (e.g., web-based interventions or videos and smartphone apps) are available, environmental distractions (e.g., noisy surroundings) may pose limitations. VR has the potential to address those challenges by providing an engaging and immersive environment, potentially leading to sustained mindfulness and larger effects for reducing CRP (33).

Because humans have a limited amount of available conscious attention, VR competes for attention otherwise directed toward interpreting stimulation from the real environment, leaving lesser cognitive capacity available for processing external distractions (31, 33). It is also possible that VR-guided mindfulness (VRGM) can help participants self-initiate and continue mindfulness practice, as well as maintain conscious and cognitive efforts required for effective practice, all of which have previously been identified as barriers to mindfulness meditation practice, particularly among novice meditators (30, 33).

VRGM has the potential to overcome some of the limitations of traditional mindfulness-based interventions, such as MBSR, that involve an instructor within a group setting. External distractions from other group members may pose limitations required for effective mindfulness practice. Regularly attending sessions may be difficult for cancer patients and survivors with chronic pain due to pain related interference with daily activity, hospitalizations, or cancer treatments, in addition to personal commitments. The timing and frequency of sessions may not meet the meditative needs of participants. For example, some may prefer morning meditations to energize oneself, while others may prefer evening meditations to relax before sleep. A weekly session may not suffice the needs for people with cancer experiencing chronic pain, who may desire to practice mindfulness daily or multiple times per day for pain management. Those living in remote areas may have difficulties with accessibility, as local mindfulness-based interventions may not be available. Additional accessibility barriers may occur due to costs associated with attending sessions (i.e., session fees, transportation, parking, etc.), especially in countries without universal healthcare. VRGM has the potential to overcome these challenges by eliminating external group distractions and providing more flexibility in the timing, frequency, and accessibility of mindfulness practice through a self-delivered intervention.

In the last 10 years, innovations in technology have dramatically driven down costs for development and implementation of VR, which has given rise to its commercialization and affordability as a cost-effective tool for clinical practice (34–36). For example, in the 1990s VR systems used in clinical research could cost up to $90,000 USD, and today in 2024 the Meta Quest 2 standalone VR headset can be purchased for $250 USD (37). Major milestones for VR technology occurred in 2016 when companies in the technology industry began mass producing VR headsets and in 2019 when the industry shifted towards the development of standalone VR headsets (a wireless, easy to use, self-contained VR system not requiring an external computer to run the software) (34). Continued investment in VR technology by big tech companies, such as Meta, Apple, Microsoft, and Google, driven by competition for potentially new lucrative VR-related markets, have resulted in a considerable increase in the image quality and immersive experience year-over-year, while continuously improving affordability and availability (34). However, the current cost of a VR headset may still be financially constraining for some, and as such an ideal scenario to improve accessibility would have cancer support centres purchase a small number of VR headsets for people with cancer to use freely within the centre.

Despite its potential benefits for patients with chronic CRP, VRGM has received relatively limited attention in the VR literature. VR has primarily been used as a distraction technique for acute procedural pain relief, with some studies incorporating video games or virtual traveling into the VR experience as a way to enhance psychological distraction and reduce self-reported pain (31). Importantly, very few studies targeting chronic pain used VR as a medium to facilitate mindfulness meditation, which is less about distraction and more about awareness of current experience. A 2015 RCT of 13 participants (33) found VRGM to be superior to a mindfulness audio track alone in reducing chronic noncancer pain symptoms. Similarly, in a 2017 single-group study (38) evaluating the benefits of VRGM for 18 participants with chronic pain (16 patients with cancer and 2 patients with neurological conditions), all participants reported a reduction in pain intensity, ranging from a 20% to a 100% reduction.

Taken together, while VRGM could be a novel promising approach for chronic CRP management, additional research is needed to evaluate its efficacy and determine its acceptability and feasibility to guide the design and implementation of future trials. Research is also needed to understand the potential mechanisms of mindfulness-related improvement in pain intensity and pain interference and to test VRGM models that are self-deliverable and home-based to enhance effectiveness and sustainability. As a first step in a program of research, this mixed methods single-group feasibility study will evaluate a home-based 6-week VRGM intervention for cancer survivors with CRP lasting longer than 3 months. The following paragraphs outline the specific study objectives.

### 1.2 Primary objective

#### 1.2.1 Objective 1: to evaluate the feasibility of a self-administered, at-home, 6-week VRGM program for chronic CRP

Feasibility outcomes include recruitment rates, intervention adherence, and study completion rates, as well as intervention safety as determined by the occurrence, type, and severity of negative side effects.

### 1.3 Secondary objectives

#### 1.3.1 Objective 2: to explore the potential benefits of a 6-week VRGM program for patients with chronic CRP

To address this proof-of-concept objective, effect sizes and confidence intervals of changes in outcome measures (pain, sleep, depressive and anxiety symptoms, fatigue, quality of life, and analgesic use) will be described across different time points.

#### 1.3.2 Objective 3: to explore potential factors contributing to the benefits of mindfulness practice for patients with chronic CRP

Specifically, pre- to post-VRGM changes in pain will be described in relation to levels of immersion and presence in the VR experience, trait mindfulness, and amount of VRGM practice.

#### 1.3.3 Objective 4: to understand cancer survivors’ experiences with VRGM

Participants will take part in in-depth, semistructured interviews to evaluate the acceptability and perceived benefits of VRGM, and to identify obstacles and facilitators of adherence and the challenges faced in initiating and maintaining VRGM practice.

## 2 Methods

### 2.1 Design

#### 2.1.1 Overview

This study will employ a prospective, single-arm, pretest-posttest, mixed methods design. Adults with chronic CRP will participate in a 6-week home-based VRGM intervention with a one-to-one interview upon intervention completion.

### 2.2 Participants

#### 2.2.1 Sample size

This protocol is designed as a mixed methods feasibility study. The recruitment goal is 15 participants, a sample which is comparable to other mindfulness studies primarily focusing on feasibility, reporting sample sizes of 13–19 participants (39, 40). This sample size can allow the evaluation of feasibility outcomes by providing crucial information about the ability to recruit this population to such an intervention, intervention safety, adherence, and study completion rates. Feasibility will also partly be determined by participants’ attitudes toward VRGM and level of satisfaction with the intervention during the semistructured interviews.

#### 2.2.2 Eligibility criteria

Patients with any type or stage of cancer, who have reported chronic CRP (i.e., for 3 months or longer), are eligible to participate. Patients could be on active treatment, posttreatment, or receiving palliative care (see Table 1 for rationale of all inclusion and exclusion criteria).

**Table 1:** Eligibility criteria.

Criteria	Rationale
Inclusion	
Chronic cancer-related pain. Specifically, one screening question will be used: “Do you have bodily pain related to cancer or as a result of cancer treatment that has lasted for more than three months?” (Answer must be yes to proceed with eligibility assessment)	This criterion is consistent with the chronic pain definition by the International Association for the Study of Pain (2).
Moderate to severe pain interference with function, as indicated by a score of >3 on the Brief Pain Inventory-pain interference with daily activities 10-point Likert scale (0 = “does not interfere” and 10 = “interferes completely”) (49)	Interventions targeting chronic pain often aim to improve both pain intensity and pain-related functional impairment (16).
Cancer diagnosis (all stages)	This criterion is intentionally broad to enhance generalizability.
Ability to speak and read in English	Mindfulness audio narrative and study assessments will be in English.
Exclusion	
Patients with dementia, Alzheimer disease, intellectual disability, amnesia, schizophrenia, psychosis, or with a history of seizures or epilepsy	People with pre-existing neuropsychological conditions may be at high risk for adverse events (e.g., confusing virtual reality with real world) (50).
Severe vision or hearing problems	This study involves prerecorded audio instructions of mindfulness techniques and a 3D virtual reality experience, requiring adequate visual and hearing ability.
Recent change (i.e., in the 2 weeks prior to study enrollment) or a planned change in opioids or antidepressants during the 6-week study period	Pain and antidepressant medications can affect pain scores during the study. While this feasibility study does not aim to evaluate efficacy, inclusion criteria are meant to be comparable to those that would be used in an efficacy trial.

### 2.3 Procedures

#### 2.3.1 Recruitment

The main recruitment strategy will be via oncology physician referral in the outpatient clinics of the Tom Baker Cancer Centre, a tertiary care teaching hospital affiliated with the University of Calgary (Calgary, Alberta, Canada). Social media platforms, such as Facebook, Instagram, and Twitter, and community cancer support organizations will also be used to enrich recruitment and reach underrepresented populations. The study coordinator will make a telephone call to referred patients or those who have expressed interest via advertisements to describe the purpose of the study, provide details about the intervention and the number and frequency of assessments to be completed, and screen for eligibility. Those who are interested in participating and eligible will sign an electronic consent form.

#### 2.3.2 Baseline call

Consenting participants will be mailed or delivered a head-mounted VR display headset (Pico Neo 3 Pro) with the VRGM protocol preinstalled. Prior to starting the intervention, participants will complete a 20 min baseline in person or by videoconference call with the investigative team to ensure receipt of the VRGM kit and for an orientation session (e.g., how to wear and set up the headset, what are the buttons required, and what they do). After orientation, participants will complete a 15 min trial session with the study coordinator or a trained research assistant (RA) to assess how comfortable they are with the VR experience and if they develop simulator sickness, which is a possible side effect of VR (41). Simulator sickness is generally attributed to the discrepancy between simulated visual motion and the sense of movement stemming from the vestibular system, producing symptoms similar to motion sickness (41). After the trial session, participants will complete the Simulator Sickness Questionnaire (SSQ) (41) with the study coordinator or RA, a well-validated scale that assesses 16 symptoms on a 4-point scale (scores 0–3): nausea, general discomfort, stomach awareness, sweating, increased salivation, vertigo, burping, difficulty concentrating, difficulty focusing, eyestrain, fatigue, headache, blurred vision, dizziness with eyes open, dizziness with eyes closed, and fullness of head. A total SSQ score greater than 20 indicates that symptoms may be concerning (41), and thus participants may have high tendency to develop simulator sickness. Therefore, participants who score >20 on the SSQ will be deemed ineligible to proceed with study participation. They will be asked to stop using the VR headset and return it to investigators. Instead, they will be provided with resources for other mindfulness platforms.

#### 2.3.3 Data collection

Consenting participants will be emailed a link before the baseline call to complete the pre- and poststudy surveys in the REDCap secure database. Daily diaries will be returned using a stamped, self-addressed envelope to the investigative team. With regard to qualitative data, semistructured interviews will be conducted in person or through videoconference call by a member of the research team using a pre-established set of questions (see Table 2) that cover the following areas: perceptions of VRGM, perceived benefits of VRGM, whether and how VRGM has impacted pain severity and interference with function, perception of pain and previous mindfulness meditation experience (42), and factors underlying satisfaction and dissatisfaction with study participation. Participants who drop out of the study will also be invited to participate in the semistructured interview. Interviews will be audio-recorded, and recordings will be transcribed verbatim.

**Table 2:** Semistructured interview script.

• Please describe your experience in the virtual reality guided mindfulness program. Was there anything outstanding for you? • Were the mindfulness techniques that you used or learned through virtual reality helpful for your pain? If so, could you please describe them and explain how they related to your day-to-day pain symptoms? • Please describe your experiences of doing virtual reality guided meditation at home. Were there strategies that you used to help with home practice? If so, could you please describe them and explain how you applied them? • Do you have any suggestions for how to improve participant experience in future studies?

### 2.4 Intervention

Consistent with previous similar studies (33, 43), the VRGM intervention will consist of a 10–15 min, once-daily session for 6 weeks. The VR headset will present a range of preinstalled 3D high-quality calming virtual environments (i.e., nature scenes and sounds), such as mountain meadows, white sands, spring creek, and other beautiful nature scenery (e.g., beaches, waterfalls, and tall trees). The headset includes a motion tracker that measures the position of the head and adjusts the visual image accordingly, which can make participants feel as if they can look around and move through the virtual environment. Built-in speakers on the VR headset will provide an audio narrative of guided mindfulness practice.

The content of the audio is based on the research team's previous work with the evidence-based mindfulness-based cancer recovery program (17) and will include instructions to perform weekly mindfulness exercises (one exercise per day) for 6 weeks (see Table 3). Each meditation exercise has its own unique preinstalled virtual environment, selected on the basis of which was best suited for each type of meditation. All virtual environments will also be customized to synchronize with the meditation audio (e.g., mixing movement with sound).

**Table 3:** Virtual reality-guided mindfulness for pain curriculum.

Day	Teaching (∼5 min; first 2 days only; can be listened to repeatedly or skipped after week 1)	Guided meditation practice (∼10–15 min)	Virtual reality experience
1	Introduction: What is mindfulness?	Awareness of breath	White sands
2	Awareness of pain	Body scan	Waterfall cliffs
3	N/A^a^	Awareness of pain	Misty summit
4	N/A	Loving kindness	Spring creek
5	N/A	Mini breathing exercises	Oceanview
6	N/A	Mountain meditation	Mountain meadows
7	N/A	Healing from pain	Forest stream

^a^ N/A, not applicable.

### 2.5 VR hardware specifications

The Pico Neo 3 Pro (PN3P) is a 6 degrees of freedom standalone VR headset with a high resolution 4k liquid crystal display (1,832 × 1,920 pixels per eye) and 90 Hz refresh rate (44). The PN3P was released in May 2021 and retailed for $699 USD. It accommodates for a wide range of different eye features, as the user is able to adjust the lens spacing at three settings (58 mm, 63.5 mm, and 69 mm) to line up with the user's interpupillary distance to ensure image clarity and allow for a more immersive experience (45). The PN3P is also suitable for a wide range of head shapes and facial features, as it uses an adjustable rear strap and polyurethane leather foam face cushion to properly position the VR device on the user's face (45). The all-hygienic materials of the P3NP make the VR headset easy to clean and ideal to share between multiple users (45). It is ergonomically designed for users to comfortably wear for long periods of time by counterbalancing the weight of the front display with the rear battery (44). The total weight of the PN3P is 673 g (46), which is in the middle range for other 4k standalone VR headsets with similar specifications and release date, such as the Oculus Quest 2 at 503 g (47) and HTC Vive Focus 3 at 785 g (48). Non-standalone (tethered) VR headsets require a cable connection to a personal computer (PC) to run the VR software, which increases the weight of the VR headset, restricts user mobility, and can be cumbersome for cable management. Tethered VR headsets also increase the complexity in setup for users and have additional costly and bulky hardware requirements (PC, graphics card, etc.), thus making a standalone system the preferred choice for a home-based VRGM intervention.

### 2.6 Feasibility evaluation

A log containing information on the number of patients approached for participation, the number referred to the research team, the number successfully contacted by the research team, and those who consented will be maintained. The number of patients who declined or did not meet the eligibility criteria and the underlying reasons will also be recorded. Another log will be maintained for the number of participants who dropped out during the study. Home practice will be tracked through engagement data from the VR device, which include session length, total time spent using the headset, and number of daily VRGM sessions. Data collected from the VR device will be verified through a daily home practice log. The log will provide space to input the date, number of VRGM sessions completed each day, total number of minutes per session, and comments about their experience and side effects. Participants will also be contacted once weekly to ask whether they have experienced any side effects. Data obtained from study logs will be used to calculate recruitment and study completion rates. Data collected from the VR system, home practice logs, and weekly emails will be used to evaluate intervention adherence and safety.

### 2.7 Outcome measures

All the study measures are described in Table 4 (51–65). Standardized measures of pain, sleep, depressive and anxiety symptoms, fatigue, and quality of life (objective 2), as well as trait mindfulness and levels of immersion and presence in the VR experience (objective 3), will be used. Sociodemographic and medical information collected from participants at baseline will include age, sex, gender, race and ethnicity, cancer type and stage, time since diagnosis, time since last treatment, presence and severity of comorbidities, educational level, marital status, occupational status, and type and frequency of nonpharmacological interventions used for pain in the last 3 months. The prescribed analgesic regimen (standing and as needed) will also be obtained. This will include the number of analgesics prescribed, types of analgesics, frequency of administration, route of administration, and prescribed dose. The entire battery of questionnaires has been user-tested and requires approximately 30 min to complete.

**Table 4:** Outcome measures.

Construct	Measure	Description
Pain		
Pain intensity	Numeric pain rating scale (51, 52)	0–10 Likert Scale (0: no pain at all; 10: worst possible pain); widely used and validated in oncology (51, 52)
Pain severity and interference	Pain questionnaire (53, 54)	7-item scale that assesses pain frequency, location, duration, intensity, and emotional upset due to pain in the past 7 days. This scale has demonstrated good validity and reliability coefficients (53, 54).
Pain catastrophizing	Pain catastrophizing scale (55)	13-item questionnaire that assesses on a 5-point scale, ranging from 0 (not at all) to 4 (always), to which degree one experiences certain thoughts or feelings during pain over the past 7 days. Studies have supported the psychometrics of this scale (55).
Neuropathic pain quality	PROMIS^a^ Neuropathic Pain Quality (56)	5-item scale that assesses neuropathic pain over the past 7 days; used to distinguish between neuropathic and non-neuropathic pain conditions. PROMIS-Pain Quality-Neuro has demonstrated good psychometric properties (Cronbach's alpha = 0.87) (56).
Pain interference	PROMIS-SF^b^-8a-pain interference (57)	8-item scale that assesses pain interference with function in the past 7 days. Items are scored on a 1–5 scale. Alpha reliability ranges from .96 to .99 and construct validity (57).
Analgesic use	Investigator-developed	Daily analgesic use log including the name, dosage, and frequency of all pain medications.
Virtual reality experience		
Immersion	1–10 likert scale add-on to the immersive experience questionnaire (IEQ) (58)	One 1–10 Likert scale that rates how immersed did a participant feel during VR (1 = not at all immersed, 10 = very immersed). The psychometrics of the IEQ questionnaire has been evaluated in a general population study of 260 participants with high reliability and validity coefficients (58).
Presence	Slater-usoh-steed (SUS) questionnaire (59)	3-item 1–7 Likert-scale questionnaire that evaluates presence (i.e., the sense of being in a VR; the extent to which a VR feels real; and the extent to which a VR is thought of as a place visited). The SUS questionnaire has demonstrated good psychometric properties in various populations with well-established concurrent validity with other measures of VR presence (59).
Sleep		
Sleep quality	Pittsburgh sleep quality index (PSQI) (60)	PSQI includes 19 items grouped into 7 subscales: (1) sleep quality, (2) sleep efficiency, (3) daytime dysfunction, (4) sleep latency, (5) sleep disturbances, (6) sleep duration, and (7) use of sleep medication. Items are scored on a 0–3 scale. PSQI has demonstrated adequate reliability and validity among cancer patients, with Alpha reliability coefficients ranging from .70 to .80 (60).
Sleep amount and quality, pain interference with sleep	Sleep diary (61)	A sleep diary that is well-validated for use with chronic pain patients. It assesses one's previous night's sleep, including the number of hours slept, sleep onset latency, number of awakenings, sleep quality (Likert scale; 0 = “extremely poor” to 5 = “extremely good”), and sleep restfulness (Likert scale; 0 = “not at all rested” to 5 = “well rested”) (61), as well as one's level of pain at bedtime and upon awakening on a 6-point Likert scale (0: no pain at all; 5: very intense pain) (61, 62)
Depression and anxiety symptoms		
Anxiety	PROMIS-SF-8a-anxiety (63)	8-item scale that measures anxiety over the past 7 days. Items are scored on a 1–5 scale. PROMIS-SF-anxiety scale has shown excellent convergent and discriminate validity with other measures, with a Cronbach's alpha of .95 (63).
Depression	PROMIS-SF-8a-depression (63)	8-item questionnaire that assesses depression over the past 7 days. Items are scored on a 1–5 scale. The PROMIS-SF-depression has demonstrated good validity and reliability coefficients (Cronbach's alpha = .91) (63).
Fatigue		
Fatigue	PROMIS-SF-8a-fatigue (63)	8-item scale that measures fatigue over the past 7 days. Items are scored on a 1–5 scale. PROMIS-SF-fatigue has demonstrated good psychometric properties with reliability coefficients ranging from .72 to .88 and concurrent validity with other measures of fatigue, ranging from 0.6 to 0.85 (63).
Trait mindfulness		
Trait mindfulness	Five-facet mindfulness questionnaire short form (FFMQSF) (64)	15-item measure of trait mindfulness with five subscales: non-reactivity to inner experience, observing sensations, acting with awareness, describing with words, and non-judging of experience. Items are scored on a 1–5 scale. The FFMQSF is widely used in oncology with sound psychometrics (64).
Quality of life		
Overall health and QOL	Two items add-on to the EORTC QLQ-C30^c^ (65)	Two 1–7 likert scales add-on to the EORTC QLQ-C30 that rates self-perceived health and QOL during the last 7 days. EORTC QLQ-C30 has been well validated in oncology (65).

^a^ PROMIS, patient-reported outcomes measurement information system.

^b^ PROMIS-SF, patient-reported outcomes measurement information system-short form.

^c^ EORTC QLQ-C30, European organization for the research and treatment of cancer (EORTC) quality of life questionnaire; QOL, quality of life.

### 2.8 Timing of assessments

Timing of assessments is fully described in Table 5 (schedule of measures).

**Table 5:** Schedule of measures.

		Treatment and follow-up							
	Enrollment	Baseline	1 week	2 weeks	3 weeks	4 weeks	5 weeks	6 weeks	12 weeks
Education and consent	X								
Screening checklist	X								
Demographic questionnaire and medication history	X								
Objective 1 (feasibility)									
Home practice log			D^a^	D	D	D	D	D	
Objectives 2 and 3 (benefits and factors)									
Pain Numeric rating scale (scores 0–10)^b^			D	D	D	D	D	D	
Immersive Experience Questionnaire^b^			D	D	D	D	D	D	
Slater-Usoh-Steed scale^b^			D	D	D	D	D	D	
Analgesic use log			D	D	D	D	D	D	
Sleep diary^c^		X						X	X
PROMIS^d^-Short form–pain interference scale		X	X	X	X	X	X	X	X
Pain questionnaire		X						X	X
Pain catastrophizing scale		X						X	X
PROMIS neuropathic pain quality		X						X	X
Pittsburgh sleep quality index		X						X	X
PROMIS-short form–anxiety scale (8a)		X						X	X
PROMIS-short form–depression scale (8a)		X						X	X
PROMIS-short form–fatigue scale (8a)		X						X	X
EORTC QLQ-C30^e^ (2 likert scales with scores of 0–10)		X						X	X
Five-facet mindfulness questionnaire–short form		X						X	X

^a^ D: daily completion over the course of the entire study through week 6.

^b^ Responses on the Pain Numeric Rating Scale, Immersive Experience Questionnaire, and Slater Usoh-Steed Scale are recorded using the virtual reality device.

^c^ Participants will maintain a sleep diary for 1 week at baseline, post intervention, and 6 weeks post intervention (12 weeks).

^d^ PROMIS, patient-reported outcomes measurement information system.

^e^ EORTC QLQ-C30, European organization for the research and treatment of cancer quality of life questionnaire.

#### 2.8.1 Objective 2

Psychosocial outcome measures (i.e., pain, sleep, fatigue, depressive and anxiety symptoms, and quality of life) will be assessed before and after the intervention and 6 weeks post intervention (follow-up). As noted above, pain-related functional impairment is often a priority in chronic pain mitigation efforts and is thus an important outcome for this feasibility trial. Therefore, pain interference with function will be assessed weekly during the 6-week intervention period and then again at follow-up. The VR device will record the intensity of participants’ pain on a Numeric Rating Scale (scores of 0–10) (51) before and immediately after each VRGM session. Participants will maintain analgesic use in the home practice logs to record their use of pain medications and dosages daily, until completion of the 6-week VRGM intervention. Additionally, they will maintain a sleep diary for 1 week before and after the intervention, as well as at 6 weeks post intervention (follow-up), including assessments of sleep duration and quality and pain interference with sleep. Since sleep diaries evaluate sleep on a daily basis, many of the limitations of retrospective self-reported sleep measures are minimized, such as recall bias and recency bias (tendency to recall last few nights) (66).

#### 2.8.2 Objective 3

The VR device will record participants’ level of immersion and presence in each VRGM session. Trait mindfulness will be evaluated before and after the intervention and 6 weeks post intervention (follow-up).

### 2.9 Data analysis

This is a small feasibility study; therefore, analyses will be descriptive.

#### 2.9.1 Quantitative analysis

Descriptive statistics will be used to calculate accrual rates (and reasons for not consenting to the trial), retention in the trial, intervention adherence, questionnaire completion, and adverse event rates (objective 1). Means and SDs will be calculated for continuous variables and frequency distributions for categorical variables. The profile of each outcome (means and SDs) will be displayed to visualize the pattern of changes over time (objective 2). Effect sizes and confidence intervals of changes in scores for outcome measures (i.e., pain, sleep, fatigue, depressive and anxiety symptoms, and quality of life) will be described across different time points (objective 2). Exploratory analyses will be used to describe the strength of associations of pre- to post-VRGM pain changes with the levels of immersion and presence in the VR experience, total amount of VRGM practice, and change in pre- to post-VRGM trait mindfulness (objective 3).

#### 2.9.2 Qualitative analysis (objective 4)

Qualitative data will be analyzed using the principles of Thematic Analysis (67), which is a rigorous inductive approach to identify and describe implicit and explicit patterns within qualitative data. The steps of Thematic Analysis (67) will be applied, including (1) reading the transcripts actively and repeatedly to become familiar with the depth and breadth of the content, (2) identifying patterns and developing initial codes (subthemes), (3) searching for and identifying overarching themes, (4) comparing and contrasting emergent themes within and across transcripts, and (5) developing and reviewing the thematic scheme. A number of strategies will be used to improve methodological rigor. To minimize researcher bias, a self-reflexivity journal will be maintained during data collection and analysis. Two members of the research team will independently code all of the transcripts and develop interim themes. Discrepancies will be discussed and resolved by consensus.

### 2.10 Ethics approval

This research has been approved by the Health Research Ethics Board of Alberta Cancer Committee (HREBA.CC-20-0411). Potential participants will be informed of study details, including confidentiality and the voluntary nature of participation, risks and benefits, and study requirements prior to signing the consent form. Participants will be made aware of their right to withdraw at any time, and that this will not affect their future care. As previously noted, VR technology is known to be safe, but it may be associated with simulator sickness. Measures are in place to minimize this risk as outlined in the protocol. The potential benefits will likely outweigh the side effects that may result from VR technology. Although the efficacy of mindfulness practices for reducing chronic CRP is an outstanding issue when delivered through an immersive virtual environment, in general, the mental and physical health benefits of mindfulness are well documented.

## 3 Discussion

### 3.1 Strengths and limitations of this study

A strength of this study is employing a mixed methods design, which can enhance the rigor of the study by including findings from different methodological perspectives. This study has and will continue to involve patient research partners throughout the project to guide research questions, design, and implementation, and add context to the research findings. This study will provide the foundation needed to design and conduct a larger study to examine the efficacy of VRGM in improving chronic CRP. A limitation of this study is its single-group design, which is necessary to evaluate the acceptability of VRGM by as many cancer survivors as possible. This feasibility study is not statistically powered to evaluate the intervention efficacy for clinically relevant outcomes.

### 3.2 Dissemination

The results will be widely disseminated to stakeholders through presentations at national and international conferences, publications in open access scientific journals, social media platforms (e.g., Twitter, LinkedIn, ResearchGate, and Academia.edu), and webinars and discussion groups through patient advocacy networks and professional oncology organizations.

## 4 Conclusions

Survivors of cancer experience increased levels of psychosocial symptoms and pain interference. This novel intervention provides a potential alternative treatment to opioid analgesics. Results from this study may inform future larger VGRM trials for chronic CRP to help reduce suffering in people with cancer.

## Data Availability

The original contributions presented in the study are included in the article/Supplementary Material, further inquiries can be directed to the corresponding author.
